# Knowledge Gaps in Biophysical Changes After Powered Robotic Exoskeleton Walking by Individuals With Spinal Cord Injury—A Scoping Review

**DOI:** 10.3389/fneur.2022.792295

**Published:** 2022-03-10

**Authors:** Christopher C. H. Yip, Chor-Yin Lam, Kenneth M. C. Cheung, Yat Wa Wong, Paul A. Koljonen

**Affiliations:** ^1^Li Ka Shing Faculty of Medicine, The University of Hong Kong, Pokfulam, Hong Kong SAR, China; ^2^Department of Orthopaedics and Traumatology, Li Ka Shing Faculty of Medicine, The University of Hong Kong, Pokfulam, Hong Kong SAR, China; ^3^Department of Orthopaedics and Traumatology, Maclehose Medical Rehabilitation Centre, Hong Kong West Cluster, Hospital Authority, Kowloon, Hong Kong SAR, China

**Keywords:** spinal cord injury, rehabilitation, exoskeleton, paraplegia, scoping review

## Abstract

In addition to helping individuals with spinal cord injury (SCI) regain the ability to ambulate, the rapidly evolving capabilities of robotic exoskeletons provide an array of secondary biophysical benefits which can reduce the complications resulting from prolonged immobilization. The proposed benefits of increased life-long over-ground walking capacity include improved upper body muscular fitness, improved circulatory response, improved bowel movement regularity, and reduced pain and spasticity. Beyond the positive changes related to physical and biological function, exoskeletons have been suggested to improve SCI individuals' quality of life (QOL) by allowing increased participation in day-to-day activities. Most of the currently available studies that have reported on the impact of exoskeletons on the QOL and prevention of secondary health complications on individuals with SCI, are of small scale and are heterogeneous in nature. Moreover, few meta-analyses and reviews have attempted to consolidate the dispersed data to reach more definitive conclusions of the effects of exoskeleton use. This scoping review seeks to provide an overview on the known effects of overground exoskeleton use, on the prevention of secondary health complications, changes to the QOL, and their effect on the independence of SCI individuals in the community settings. Moreover, the intent of the review is to identify gaps in the literature currently available, and to make recommendations on focus study areas and methods for future investigations.

## Introduction

### Background

To many, spinal cord injury (SCI) is the most commonly associated with the loss of ability to ambulate and experience external stimuli, or perhaps the loss bowel and urinary function. However, there are many additional secondary health complications that can manifest as a result of chronic SCI—these include (and are not limited to) cardiovascular (CVS) disease, an increased risk of osteoporosis and fractures, urinary and bowel complications, pressure injuries, spasticity, and neuropathic pain syndromes (1, 2). Such comorbidities significantly reduce the quality of life (QOL) and increase the risks of premature mortality for individuals with SCI.

Rehabilitation plays a key role in the management of spinal disorders, such as SCI (3). Research has shown that time spent upright, either standing or walking, is beneficial for the prevention and treatment of secondary health complications of SCI (4–7). Early attempts at solving problems associated with immobility in SCI included leg braces and orthoses used to assist standing and walking (4–6). Although these devices did prove to somewhat fulfill their intended design purposes, the increased energy expenditure from truncal and upper limb muscles, slow ambulation speed, and safety concerns, have discouraged some wheelchair users from converting to leg braces (4). More recently, exoskeletons have been developed for individuals with SCI as an alternative for assisting ambulation—both for the purposes of rehabilitation and long-term health maintenance.

### Definition of Exoskeletons

The term exoskeleton encompasses a wide range of devices with differing applications. In its original iteration, an exoskeleton is a device that augments the performance of an able-bodied wearer (8). For the purposes of SCI individuals, exoskeletons provided two main functions—to facilitate early rehabilitation and assist ambulation. The first exoskeleton to be designed for SCI was the Lokomat (9). The Lokomat was one of a categories of exoskeletons collectively known as residential exoskeletons, indicating that it could only be used within fixed spaces, such as on a treadmill. These early exoskeletons have since been referred to as a driven gait orthosis, and the type of rehabilitation and gait training involving the use of these devices are collectively known as body-weight-supported treadmill training (BWSTT). Over time, the newer generations of exoskeletons were designed with increased mobility and portability. These devices, known as overground exoskeletons, are designed with portable systems that allow the user to ambulate freely indoors and outdoors, and have provided clinicians and patients with a useful tool to increase the life-long over-ground walking capacity of individuals with SCI. Currently, exoskeletons can be subdivided into assistive devices to be used in the community, such as the Suitx Phoenix, Indego Personal, and Rewalk exoskeletons; or devices designed for rehabilitation with a therapeutic intent, such as the EksoNR and Indego Therapy exoskeletons (8).

### Proposed Benefits of Exoskeleton Use

Beyond the benefits related to physical and biological function, exoskeletons have been suggested to improve the SCI individuals' QOL by enabling their participation in day-to-day activities (10, 11). In a recent survey among individuals with SCI, the restoration of walking function was seen as the highest priority (12); another study had previously commented that other functions, such as improved bladder and bowel function, and the elimination of autonomic dysreflexia, were found to be of greater priority than walking in another group of individuals of SCI (13). We believe that the health priorities of SCI individuals are likely to be culturally-dependent, and the results of studies on this may vary according to study design and outcome metrics chosen. Furthermore, a meta-analysis on the clinical effectiveness and safety of exoskeletons has suggested that the potential benefits provided by exoskeleton use may offset the costs associated with the management of secondary ailments resulting from SCI, thereby resulting in a net reduction in costs to the healthcare systems (14).

## Objectives of This Scoping Review

Most of the currently available studies have reported on the impact of exoskeletons on the QOL and prevention of secondary health complications, on individuals with SCI, are of small scale and are heterogeneous in nature. Moreover, few meta-analyses and reviews have attempted to consolidate the dispersed data to reach more definitive conclusions of the effects of exoskeleton use (14–19). Many of these reviews included other forms of gait training, such as BWSTT (19), or robotics, such as exoskeletons for upper limbs (16), while others have included the evaluation of exoskeletons beyond individuals with SCI, such as those with a history of stroke (18). Furthermore, many studies have used the term exoskeleton loosely, often applying the term to different devices, such as reciprocating gait orthosis (RGO), driven gait orthoses, bodyweight-supported treadmill trainers, and some leg braces.

This scoping review seeks to provide an overview on the known effects of overground exoskeleton use, on the prevention of secondary health complications, changes to the QOL, and the effects on the independence of SCI individuals in community settings. The intent of the review is to identify gaps in the literature currently available, and to make recommendations on focus study areas and methods for future investigations.

This review shall focus on data on the newer generation, overground, lower-limb, and powered robotic exoskeletons (referred from this point onward as exoskeletons).

## Materials and Methods

### Scoping Review

The reporting of methods and results in this scoping review follow the Preferred Reporting Items for Systematic reviews and Meta-Analyses extension for Scoping Reviews (PRISMA-ScR) guidelines, and a PRISMA-ScR checklist can be found in Appendix 1. The framework by Arksey and O'Malley (20) has been referenced for the protocol of this scoping review. The summarized protocol of this scoping review can be found in Appendix 2.

### Data Sources and Search Strategy

PubMed, Cochrane, and EMBASE were searched for relevant articles from database inception to June 19, 2021; the bibliographies of included articles were screened. The search term used included “exoskeleton,” “spinal cord injury,” and related keywords, both as the free-text keywords and controlled vocabulary of their respective databases (e.g., MeSH for PubMed and Cochrane Library). The details of the search terms used for each database are included in Appendix 3. There was no gray literature included in our search strategy due to the large number of results generated from our search on peer-reviewed platforms. The refinement of protocol was done after the initial search, with focus placed on a defining eligibility criterion with specific attention to the list of biophysical characteristics to include (mentioned below).

### Eligibility and Exclusion Criteria

The inclusion criteria were as follows: (1) English language; (2) peer-reviewed journals; (3) studies from the inception of respective databases, up to June 26, 2021; (4) human studies; (5) SCI that caused walking disabilities; 6) the use of over-ground exoskeleton for rehabilitation, specifically as walking aid; (7) the outcome metrics of a predefined list with biophysical characteristic known to affect SCI individuals (cardiovascular [CVS], bowel, body composition, bone mass density [BMD], fracture rate, pain, spasticity, QOL, and independence and community applicability). The exclusion criteria were as follows: (1) non-SCI; (2) concomitant brain injury, neurological injury, or other cognitive impairments; (3) the use of other gait training devices (i.e., driven gait orthoses and BWSTT), without the use of over-ground exoskeleton; (4) outcome metrics that do not include any one from the predefined list of biophysical characteristics; (5) non-English language; (6) non-clinical trial; (7) the use of devices not for gait training; (8) non-human studies; (9) clinical trial in-progress; (10) pediatric study; (11) full-text inaccessible; and (12) review article.

### Study Selection

EndNote (X9) was used as the citation software to store all the studies from the initial search. Duplicates were initially removed using the automatic function in EndNote, with remaining duplicates removed manually through Excel (by 2 reviewers independently). Studies were first screened through their titles and abstracts and followed by full-text screening.

### Data Extraction and Charting

The final selection of studies was separated into individual groups based on the type of biophysical characteristic that was evaluated; studies that measured more than one biophysical characteristic were included more than once across different tables. Data extraction templates were generated *a priori*, with minor modifications done after the initial search, and the data elements extracted included: study, intervention, number of training sessions, ASIA grade, number of subjects, time-points during trial when specific relevant measurements were taken, outcome metrics used in the study, and the presence of any control arms.

## Results

In total, 654 articles were found in the initial search, 231 of which were found to be duplicate; 329 articles were excluded during the screening of the titles and abstracts, and a further 52 were eliminated after screening the full texts. In the final analysis, 42 papers were included from the initial search, combined with an additional 8 from scanning through the reference lists of relevant studies. Hence, 50 unique studies were included in the charting process. A flow diagram with the details of searching and screening process can be found in Appendix 4.

## CVS Health

Cardiometabolic diseases are considered as the leading cause of mortality among individuals with chronic SCI living in the community (21). The potential benefit of regular ambulation on combating secondary medical problems has been well documented previously (22, 23). A total of 31 articles related to this topic were found in the search and data extraction is listed in Table 1.

**Table 1:** Cardiovascular health.

**References**	**Intervention (Exoskeleton)**	**No. of training sessions**	**Duration of each session (mins)**	**ASIA Grade (No. of participants)**	**Point when measurements were taken**	**Metrics used**	**Presence of any controls**
Arazpour et al. (24)	Powered gait orthoses^*^	24	120	1A/4B (5)	During each session	PCI	Same study subjects undergoing the same training with knee-ankle-foot orthoses (HKAFO) and isocentric reciprocating gait orthosis (IRGO)
Asselin et al. (25)	ReWalk	40	60–90	7A/1B (8)	Test performed around 40th session	VO_2_ (during sitting, standing and walking)	No
						VO_2_pMax	
						%VO_2_R	
						HR (during sitting, standing and walking)	
						HRpMax	
						%HRR	
						RPE	
Bach Baunsgaard et al. (26)	Ekso, Ekso GT	16	^*^	36A/B/16C (52)	During each session	RPE	No
Benson et al. (27)	ReWalk	10	60	7A/3C (10)	Before and after each session	HR, BP	No
						VAS for fatigue	
Escalona et al. (28)	Ekso GT	10	28	12A/1B (13)	After training program	VO_2_, VCO_2_, VE, VT, RR, HR	No
						HRpeak, VO_2_peak	
						%HRpMax, %VO_2_peak, RER, RPE (to represent intensity of physical activity)	
Evans et al. (29)	Indego	0	^*^	5A (5)	2 test sessions on non-consecutive days	VO_2_, VO_2_peak, %VO_2_peak	No
						HRpeak	
						Walking economy = rate of oxygen consumption per distance walked (in L/m)	
						METsi	
Evans et al. (30)	Ekso GT	72	60	C/D^*^ (8)	Before, midway and after training program	HR, THBI	No
Evans et al. (31)	Ekso GT	72	60	4C/4D (8)	Before, midway and after training program	Brachial and ankle BP	8 (SCI ASIA grade 5C/3D) receiving conventional activity based training (AVT)
						PCI	
						HR, HRV	
						THBI	
Farris et al. (32)	Vanderbilt lower limb exoskeleton	1 (Walking test without training)		1A (1)	Before and after each walking test	HR	No
Faulkner et al. (33)	Ekso + Physiotherapy	10	90	4A/1B/1C (6)	Before and after training program	MAP, SBP, DBP, PP, cSBP, cDBP, cPP, HR, AP, AIx, Aix75 obtained through pulse wave analysis	6 (SCI ASIA grade 2A/2B/2C) receiving physiotherapy only
Gorgey et al. (34)	Ekso	12	60	1A (1)	Before and after each session	BP, HR	No
Hartigan et al. (35)	Indego	^*^	^*^	6A (6)	^*^	THBI	No
Jang et al. (36)	Angelegs	30	30	Motor-incomplete (1)	Before and after training program, and at 6 weeks follow up	VO_2_	No
						METs	
Juszczak et al. (11)	Indego	26	N/A	30A/5B/10C (45)	Before and after training program	RPE (indoor and outdoor)	No
Khan et al. (37)	ReWalk	52	^*^	6A/2B/3C/1D (12)	After training program	PCI	No
Kim et al. (38)	Hyundai Medical Exoskeleton [H-MEX]	30	60	7A/1B/2C (10)	Before and after training program	Spirometry	No
Knezevic et al. (39)	ReWalk	60	60	4A/1B (5)	20th, 40th, 60th session	VO_2_ (during sitting, standing, walking and recovery)	No
						RPE	
						CT	
						HR, %HRpMax	
Kozlowski et al. (40)	Ekso	20	120	3A/1B/3C (7)	Before, midway, after each session	HR, RPE, METs	No
Kressler and Domingo (41)	Ekso	1	6	Motor-incomplete (2)	Before, midway, after each session	HR, VO_2_, VCO_2_	Same study checks exercising with an arm cycle ergometer on a non-walking day
						TEE	
Kressler et al. (42)	Ekso	18	60	3A (3)	First and last session, midway through training program	VO_2_peak, TEE, HR, walking economy	No
						HOMA-IR	
						Glycaemic markers of cardiometabolic disease risk (glucose, insulin, HbA1c)	
						Lipid markers of cardiometabolic disease risk (TC, HDL, LDL, VLDL, TC-to-HDL ratio, TG)	
Kressler et al. (43)	Ekso GT	1 (Walking test without training)	^*^	Motor-incomplete (4)	After each walking test	VO_2_, VCO_2_, TEE	No
						MAP	
						RPE	
						RPP	
Kwon et al. (44)	Rewalk	20	60–90	10A (10)	Midway and after training program	HR, HRmax	Same study subjects undergoing the same training with knee-ankle-foot orthoses (KAFO)
						PCI	
						VO_2_, VO_2_peak	
						METs	
						TEE	
Maher et al. (45)	Ekso GT	2–4 weeks^*^	14	A-C^*^ (10)	4 unrandomised days in a 10-day period	VO_2_ (during sitting, standing and walking)	10 Non-SCI individuals training in exoskeletons
						VO_2_peak	
						TEE	
						Walking economy = metabolic cost of exercise (in kilocarlories/m)	
						Avg TEE during walking outdoors	
						2-hour OGTT at baseline and immediately postbionic ambulation glucose	
						HOMA2-IR	
McIntosh et al. (39)	Ekso GT	25	60	4A/5C/1D (10)	Before, midway, after each session	HR, BP, SpO_2_	No
						Modified RPE	
Pérez-Nombela et al. (46)	Exo-H2 (Technaid)	1 (Walking test without training)	^*^	1C/2D (3)	Before and after each walking test	BP, HR	No
						VO_2_	
Sale et al. (47)	Ekso	20	45	2A/1C (3)	Before and after training program	VAS for fatigue	No
	Ekso	20	45	3A/4B/1C (8)	Before and after training program	VAS for fatigue	No
						RPE (outdoors and indoors)	
Spungen et al. (48)	ReWalk	45	60–120	5A/2B (7)	Before, midway, after each session	RPE	No
Wu et al. (49)	Rewalk	120	60	1C (1)	Before and midway during walking session	HR, BP	No
Xiang et al. (50)	AIDER	16	50–60	8A/1C (9)	Before and after training program	Spirometry (FVC, FEV1, FEF25/50/75)	9 (SCI ASIA grade 4A/2B/3C) receiving conventional strength training and aerobic exercise
					During training session	HR, HRmax	
					6MWT	HR, SpO_2_	
						RPE	
Zeilig et al. (51)	ReWalk	13.7	50	A/B (6)	Before, midway, after each session	HR, BP	No
						VAS for fatigue	

* *Details not provided; N/A, Not applicable; %VO_2_R, VO_2_ during walking expressed as a percentage of oxygen uptake reserve; %HRmax, HR expressed as percentage of HRmax; %HRR, HR during walking expressed as a percentage of heart rate reserve; %VO_2_peak, oxygen consumption as a percentage of VO_2_peak; AIx, augmentation index (in %), defined as the augmentation pressure expressed as a percentage of central pulse pressure; AP, augmentation pressure; cDBP, central diastolic blood pressure; cSBP, central systolic blood pressure; CT, cost of transport (metabolic efficiency) measured by VO_2_/velocity (in m x mL/Kg); DBP, diastolic blood pressure; HOMA2-IR, homeostatic Model-2 Assessment of Insulin Resistance for insulin resistance; HR, heart rate; HRpeak, peak heart rate; HRpMax, maximum predicted heart rate; HRV, heart rate variability; MAP, mean arterial pressure (in mmHg); METs, metabolic equivalent of tasks; METssci, METs for persons with SCI; OGTT, oral glucose tolerance test; PCI, physiological cost index; PP, pulse pressure; RER, respiratory exchange ratio; RPE, rate of perceived exertion (based on the Borg scale); RPP, rate pressure product (HR x SBP); RR, respiratory rate (in cycles/min-1); SBP, systolic blood pressure; SpO_2_, peripheral oxygen saturation; TEE, total energy expenditure expended from fat and carbohydrate(CHO) oxidation; THBI, total heartbeat index; VAS, visual analogue scale; VCO_2_, CO_2_ production (in mL/min); VE, ventilation (in L/min); VO_2_, oxygen uptake (in in mL/kg/min); VO_2_peak, peak oxygen uptake; VO_2_pMax, maximum predicted VO_2_ during lower-limb exercise; Walking economy, rate of oxygen consumption per distance walked (in L/m)/ metabolic cost of exercise (in kilocarlories/m)*.

### Metabolic Equivalent of Task

One metric that has been used by multiple studies, to quantify the physical exercise associated with exoskeleton use, has been the metabolic equivalent of task (MET) (52). It is well-known that 1 MET is equal to the energy expended during quiet sitting, and in persons with SCI, 1 MET is defined as 2.7 ml/kg/min (reported as METssci) (53). One set of guideline from the American College of Sports Medicine has indicated that physical exercise maintained at 3.3 METS for 1 h/3 days/week is necessary to achieve preventative health benefits for secondary cardiometabolic conditions, where 3.0–5.9 METs equates to a moderate intensity of exercise (54). One meta-analysis by Miller et al. (14), found that it was possible to achieve an average METs of 3.3 during exoskeleton walking, thereby suggesting that exoskeleton use allows a paraplegic individual to carry out a sufficient amount of physical activity which is conducive to yield health benefits, yet without causing the early fatigue as experienced by the previous modes of ambulation (14).

### Perceived Exertion

Another method, through which the intensity of exercise has been quantified with, is the Borg's rate of perceived exertion (RPE) scale (55, 56). In the same meta-analysis by Miller et al. (14), their analysis achieved an average intensity that was marginally below the threshold for “moderate” intensity, required for acquiring the quoted health benefits. Other studies reported a wide range of RPE, varying from “very light” to “somewhat hard” (11, 28, 39, 57, 58). This variation may be due to the fact that the test subjects had different amounts of exposure to their respective exoskeletons, as novelty of the device and anxiety may contribute to a higher RPE (59). However, the confidence of RPE as a valid indicator is debated (56), as the rating can be confounded by other psychological factors, such as mood state, motivation, and exercise experience (60). On the whole, most studies found a decrease in effort and fatigue resulting from the exoskeleton use (11, 48, 57, 58), from the beginning to the end of a training program, which has been suggested to aid in increasing the user compliance with the device (11). The study by Juszczak et al. (11) found a significant decrease in RPE from baseline to the end of the study. However, this decrease was only found to be significant when walking with the exoskeletons indoors, not outdoors. This was likely due to the increased difficulty and time required to achieve a greater proficiency of the exoskeleton outdoors. We believe that it is intuitive that users consume less energy as they become more proficient with their exoskeleton.

### Gas Analysis

A more objective means of characterizing exertion is using oxygen uptake, also known as gas analysis. With the increase of physical load, oxygen demand and energy expenditure rise (61, 62). Therefore, the most ideal way of reflecting the metabolic activity is performing a full gas analysis based on the amount of oxygen consumed, represented by VO_2_, and carbon dioxide expelled, represented by VCO_2_. These parameters were utilized in several studies that we had reviewed (41–45). In particular, a case-control study on the Ekso GT device found that although the energy expenditure was greater in prolonged ambulation, compared with sitting and standing, it was still below guideline intensities necessary to experience the cardiorespiratory benefits (45), which was concordant with results from a previous study (42). Meanwhile, Kwon et al. (44) found no statistically significant difference in VO_2_ during ambulation with the Rewalk device, or with a knee-ankle-foot orthoses (KAFO), suggesting that KAFO may be a more cost-effective alternative for cardiorespiratory exercise. It is worth noting, that the measurement of gaseous exchange requires laboratory instrumentation that is not generally available in a clinical setting (32, 62). In our review, other metrics commonly utilized were the Total Heart Beat Index (THBI) (30, 31, 35) and the Physiological Cost Index (PCI) (24, 32, 37, 44). Both of these measures have been shown to be strongly correlated with oxygen uptake. This was reflected in the study by Kwon et al., where the ReWalk exoskeleton was found to have significantly greater energy efficiency than KAFO across all parameters, but VO_2_max and PCI (63). However, PCI has the advantage of only requiring a single measurement of the heart rate, rather than a continuous measurement, as necessitated by the THBI (32, 62). Studies using THBI revealed similar results to the (abovementioned) studies using gas analysis, where a decrease in THBI was observed after the first 6 weeks of training, denoting that a reduction in exercise intensity over time (30, 31).

### Other Parameters for Cardiometabolic Health

Many studies opted to use a variety of metrics in evaluating the use of exoskeletons on cardiometabolic health. Of note, the case-control study by Faulkner et al. (33) measured the augmentation index (AIx) to evaluate the CVS health of their SCI test subjects—defined as the augmentation pressure expressed as a percentage of central pulse pressure, which is supported by previous literature favoring the use of central blood pressure (BP) over peripheral BP for determining the risk for CVS events (64). In that particular study, 12 SCI individuals were separated into two groups, one which involved a 5-day exoskeleton program with the Ekso exoskeleton, combined with physiotherapy; and another group which received just physiotherapy only; the Ekso group received 60 min of conventional therapy + 90 min of exoskeleton training a day, while the control group received 60 min of conventional therapy + 60 min of home-based rehabilitation exercise session. Their results found a significant decrease in AIx in the group with the Ekso exoskeleton, but not in the control group receiving physiotherapy only. This finding was particularly relevant as a 10% absolute increase in central AIx is associated with a 32% increase in the risk of CVS events and 38% increase in all-cause mortality (64). Nonetheless, the decrease in the risk of CVS among study participants was unknown.

A case-control study by Maher et al. (45) compared the metabolic differences in SCI individuals using the Ekso GT exoskeleton, with normal non-SCI individuals without the exoskeleton. Their results found no significant difference in blood glucose and insulin (after an oral blood glucose tolerance test [OGTT]) and insulin resistance (as measured by Homeostatic Model-2 Assessment of Insulin Resistance [HOMA2-IR]) in the SCI group after 2–4 weeks of training. This was suggested to be due to the lack of impaired glucose tolerance at baseline. The average VO_2_ peak and total energy expenditure (TTE) among its participants indicated a “light” exercise intensity.

Some studies opted to evaluate pulmonary-related changes because of their implications on exercise performance (65). These studies evaluated respiratory parameters, such as forced vital capacity (FVC) and forced expiratory volume (FEV1), *via* the aid of pulmonary spirometry tests (38, 50).

Interestingly, as the proficiency of the user increases over time, combined with the increased metabolic efficiency in the newer models of overground exoskeletons, the metabolic benefits provided by exoskeleton use may be insufficient to provide the health benefits (59). The newer designs of exoskeletons which can provide variable assistance may be useful for addressing this particular problem.

## Bowel and Bladder Function

Spinal cord injury can lead to the dysfunctional bowel peristalsis, abnormal defecation patterns, and a variety of urinary voiding dysfunctions (66, 67). Most studies, among 11 found on this topic, that evaluated the effects of exoskeleton use on bowel and bladder function used self-reported data from SCI individuals (11, 40, 68), or specific questionnaires to quantify changes on bladder and bowel habit (17, 51, 69) (Table 2).

**Table 2:** Bowel and bladder function.

**References**	**Intervention (Exoskeleton)**	**No. of training sessions**	**Duration of each session (mins)**	**ASIA Grade (No. of participants)**	**Point when measurements were taken**	**Metrics used**	**Presence of any controls**
Baunsgaard et al. (16)	Ekso GT	24	31.5	36A+B/3C/16D (52)	First, last session and follow up	International SCI Basic Data Sets to assess bowel function, LUT function	No
Chun et al. (70)	ReWalk	25–63	30–90	9A/ 2B (11)	Before and after training program	Modified Lynch Gastrointestinal (GI) Survey for Patients with SCI	No
						Stool consistency using the Bristol Stool Scale (BSS)	
						SCI-QOL Bowel Management Difficulties Short Form Instrument	
Esquenazi et al. (68)	ReWalk	24	75–90	Chronic thoracic motor-complete SCI (12)	After training program	Patient self-reporting on bowel and bladder function	No
Huang et al. (66)	MBZ-CPM1 + defecation management training + manual therapy	16	20	Acute (T8-L2) incomplete (12)	Before and after training program	Glycerine enema	12 individuals with incomplete SCI training on BWSTT + defecation management training + manual therapy
						Defecation time (mins)	
Juszczak et al. (11)	Indego	26	^*^	30A/5B/10C (45)	Before each session	Patient self-reporting on bowel and bladder function	No
Kim et al. (38)	Hyundai Medical Exoskeleton [H-MEX]	30	60	7A/1B/2C (10)	Before and after training program	Colon transit time (oral enema and plain radiograph)	No
Kozlowski et al. (40)	Ekso	20	120	3A/1B/3C (7)	^*^	Patient self-reporting on bowel and bladder function	No
Platz et al. (69)	ReWalk	28–35	60	6A/1C (7)	After training program	Satisfaction questionnaire on bowel function only	No
Raab et al. (71)	Rewalk	120	60	1C (1)	^*^	^*^	No
Stothers et al. (72)	Ekso	^*^	^*^	3A (3)	^*^	Bladder diaries	Same study subjects walking with Lokomat + the same protocol for able bodies controls
						“Validated LUTS scores”	
Zeilig et al. (51)	ReWalk	13.7	50	A/B (6)	After training program	Questionnaire with Likert scale for bowel function only	No

* *Details not provided; N/A, Not applicable*.

### Subjective Effects of Exoskeleton Use

Studies that used self-reported data, all showed the mitigation of neurogenic bowel or bladder dysfunctions, though the statistical significance of the bowel habit improvements were unknown. Similarly, the studies that utilized patients questionnaires, all found no significant improvement in bowel habit.

Although some of the individual studies appear to disagree with each other, it should be noted that the results are very much dependent on the metrics chosen for evaluation. For example, in an exploratory study by Baunsgaard et al. (17), although the direct assessments of bowel function and lower urinary tract function showed insignificant changes in the International SCI Bowel and Lower Urinary Tract Basic Dataset over a 4 week follow-up period, an indirect assessment using the Spinal Cord Independence Measure (SCIM) III, showed a marked improvement on the bowel and use of toilet score.

### Objective Measurements of Defecation

In one of the case-control studies we identified on this subject, Huang et al. (66) utilized an exoskeleton on a cohort of subjects with incomplete SCI. Subjects were separated into two groups. Subjects in the interventional group received training on the ManBuZhe [Tian Jin] Rehabilitation Equipment Co., Ltd. China exoskeleton; while subjects in the control group received training on a BWSTT. Their results showed that in contrast with the control group, in the interventional group receiving exoskeleton training, there was a significant decrease in glycerin enema use and defecation time from baseline to study endpoint.

Kim et al. (38) used a similar approach by measuring the colon transit time in 10 study subjects. Simple abdominal radiographs were obtained 7 days after the administration of oral radio-opaque markers to evaluate the bowel movement. Out of the three participants with constipation at baseline, only two were found to have a reduced transits times, though one participant was found to have an increased transit time. Another objective measurement included the evaluation of stool consistency, using the Bristol Stool Scale (BSS) by Chun et al. (70). Similar results were achieved where the all but one of the participants has a BSS score of 4 or 5, indicating an ideal stool consistency.

### Objective Measurements of Bladder Function

Most studies evaluating bladder function used more subjective parameters by utilizing patient questionnaires. (11, 17, 40, 68). One exception was the study by Stohers et al. (72), where the changes in bladder function were quantified using lower urinary tract symptoms (LUTS) scoring. We did not identify studies which utilized objective measures, such as urodynamic studies to evaluate bladder function after exoskeleton training.

## Body Composition

It has been previously shown that the muscle wasting of approximately 30%, and an increase in intramuscular adipose tissue of approximately 100% occurs in individuals with SCI at as early as 6 weeks post-injury (73). These changes in body composition are likely to increase the risk of secondary metabolic complications. A review of 3 articles on this topic is listed in Table 3.

**Table 3:** Body composition.

**References**	**Exoskeleton**	**Intervention (Exoskeleton)**	**Duration of each session (mins)**	**ASIA Grade (No. of participants)**	**Point when measurements were taken**	**Metrics used**	**Presence of any controls**
Asselin et al. (74)	ReWalk	60	60	6A/2B (8)	Before and after training program	Weight, total fat, trunk fat, leg fat, arm fat, VAT (kg)	No
						Total lean, trunk lean, leg lean, arm lean (kg)	
Cirnigliaro et al. (75)	Ekso	100	60	*(8)	Before and after training program	Total body fat mass (TBFmass)	No
						Total body fat percent (TBF%)	
						Android adipose tissue percent (AAT%)	
						Visceral adipose tissue percent (VAT%)	
						Subcutaneous adipose tissue percent (SAT %)	
Karelis et al. (63)	Ekso	18	60	5A (5)	Before and after training program	BMI, body weight, height	No
						CSA of calf muscle, subcutaneous adipose tissue, intramuscular adipose tissue	
						Total fat, arm fat, leg fat, appendicular fat, trunk fat (%)	
						Total lean, arm lean, leg lean, appendicular lean, trunk lean (kg)	

*Lean, Lean body mass; VAT, visceral adipose tissue; Fat, fat body mass; CSA, cross-sectional area; BMI, body mass index*.

### Changes in Body Mass Composition

At the time of the writing of this review, only three studies have evaluated the changes on body composition in SCI individuals with exoskeleton use. One interventional study by Karelis et al. (63) utilized the Ekso device for intervention and DEXA scan for the measurement of body composition, and the study demonstrated significant improvements in body weight, body mass index (BMI), leg lean body mass (LBM), and total fat mass percentage, but not total LBM. The study showed an increase in the cross-sectional area of muscles of the calf, favoring the use of exoskeletons in maintaining healthy body compositions in individuals with SCI, including those with complete (AIS Grade A) SCI. These findings are particularly favorable for exoskeleton use, given that results were demonstrable after only 18 sessions of training.

Another recent interventional study by Asselin et al. (74) found improvements in total fat, trunk fat, and body weight in SCI individuals after 60 session of training, although there was no significant improvement in total or leg LBM. On the other hand, Cirnigliaro et al. (75) were able to elicit a significant decrease in total body fat mass, total body fat percentage, android adipose tissue percentage, and subcutaneous adipose tissue percentage in their study with eight individuals with chronic SCI, after 100 sessions of training. This may suggest that the duration of training may be a limiting factor in eliciting positive effects on body composition.

It is worth noting that some of the results from these studies are supported by similar investigations performed with BWSTT (76–78). One of such studies showed an increase in fatigue-resistant type II muscle fibers, on muscle biopsy, after 6 months of three-time-weekly training, indicating an improvement in the quality of muscle fibers and quantity (79).

## Bone Health

A decrease in BMD, at a rate of 3–4% per month, has been reported within the first year of a SCI. This can increase the risk of fractures by 25–46% (80, 81). Although static standing and resistance training alone are seen to counteract the deterioration in bone health, it has been shown that gravity-derived high-standing loads and impact accompanying low walking speeds, are the superior sources of stimuli for the maintenance of bone health (5, 7).

### BMD Changes With Exoskeleton Training

Studies evaluating the changes in BMD in SCI individuals with exoskeleton are rare (38, 63, 68). In a small-scaled interventional study by Karelis et al. (63), researchers found an insignificant increase in BMD by measuring the peripheral quantitative CT (pQCT) of the tibia among 5 SCI individuals. Despite the relatively small study size, the increase of 14.5% in BMD was seen as a particularly remarkable result given that the study subjects only received 6 weeks of training. An improvement in BMD, though not statistically significant, was also seen in the study by Kim et al. (38). Though one participant had a 13.9% increase in BMD in the right femoral neck, which improved the BMD status from osteopenia to normal. Pitfalls in related studies include the heterogeneous measurements for BMD (knee and hip or femoral neck) (82), varying training regimens, and the lack of control for medical treatment for osteoporosis. Further controlled studies are needed in this area to elucidate the actual effects of training dosage and intensity on change in BMD. Data extraction of 3 articles on this topic is listed in Table 4.

**Table 4:** Bone health.

**References**	**Intervention (Exoskeleton)**	**No. of training sessions**	**Duration of each session (mins)**	**ASIA Grade (No. of participants)**	**Point when measurements were taken**	**Metrics used**	**Presence of any controls**
Esquenazi et al. (68)	ReWalk	24	75–90	Chronic thoracic motor-complete SCI (12)	^*^	BMD at femoral neck and lumbar spine	No
Karelis et al. (63)	Ekso	18	60	5A (5)	Before and after training program	Total BMD, leg BMD, pQCTtibia BMD (g/cm^2^)	No
Kim et al. (38)	Hyundai Medical Exoskeleton [H-MEX]	30	60	7A/1B/2C (10)	Before and after training program	BMD at femoral necks bilaterally	No

* *Details not provided; BMD, bone mineral density; pQCT, peripheral quantitative computed tomography*.

## Neuropathic Pain

A systematic review by Dijerks et al. (83) reported the prevalence of chronic pain range from 26 to 96% in SCI individuals from 42 individual studies. A prevalence of 5–37% was reported for pain that was refractory to the treatment. A number of studies, among 10 found on this topic, adopted a numerical rating [i.e., visual analog scale (VAS)] for the evaluation of pain (6, 37, 42, 47, 51), whilst some others utilized self-reported data from patients, to identify changes in neuropathic pain after individual training sessions with the exoskeleton (11, 40, 68, 69) (Table 5).

**Table 5:** Neuropathic pain.

**References**	**Intervention (Exoskeleton)**	**No. of training sessions**	**Duration of each session (mins)**	**ASIA Grade (No. of participants)**	**Point when measurements were taken**	**Metrics used**	**Presence of any controls**
Baunsgaard et al. (17)	Ekso GT	24	31.5	36A+B/3C/16D (52)	After each session	International SCI Pain Basic Data Set (version 2.0)	No
Esquenazi et al. (68)	ReWalk	24	75–90	Chronic thoracic motor-complete SCI (12)	Before and after each session	Patient self-reporting	No
Juszczak et al. (11)	Indego	26	^*^	30A/5B/10C	Before each session	Patient self-reporting	
Khan et al. (37)	ReWalk	52	^*^	6A/2B/3C/1D (12)	Before and after each session	NRS	No
Kozlowski et al. (40)	Ekso	20	120	3A/1B/3C (7)	^*^	Patient self-reporting	No
Kressler et al. (42)	Ekso	18	60	3A	Before and after training program	International SCI Basic Pain Dataset (for specific pain type) + Multidimensional Pain Inventory (SCI) (for overall pain severity)	No
						Neuropathic Pain Symptom Inventory	
						VAS pain	
Platz et al. (69)	ReWalk	28–35	60	6A/1C (7)	First, last session and follow up	Patient self-reporting	No
Sale et al. (47)	Ekso	20	45	2A/1C (3)	Before, midway, after each session	VAS pain	No
Stampacchia et al. (6)	Ekso	1	40	12A/2B/7D	Before and after the session	Numerical Rating of pain	No
Zeilig et al. (51)	ReWalk	13.7	50	A/B (6)	Before, midway, after each session	VAS pain	No

* *Details not provided; NRS, numerical rating scale (0–10); VAS, visual analogue scale*.

There have been two large-scale studies on the effect of exoskeleton use on the neuropathic pain in SCI individuals, these was a multicenter trial with 52 SCI individuals training with the Ekso GT (17), and another single center with 45 SCI individuals training with the Indego exoskeleton (11). Both trials reported no statistically significant change in pain from the initiation of the intervention till follow-up, though no new pain sensations were provoked. This was attributed to be due to the floor effect, where self-reported pain was low from baseline (11). Another study with 21 SCI individuals supported this claim, where significant pain reduction was only observed in patients who reported pain before the training session (6).

Few small-scale studies found improvements in pain after training with exoskeletons (42, 47, 51). One study, where SCI individuals trained with ReWalk exoskeleton for up to 24 sessions, had 5 out of 12 patients reporting a reduction of pain immediately after a training session (68). Of note, in a case series involving 2 Asian individuals, both of them experienced severe pain requiring pain relief medications at baseline, received 60 training sessions with the hybrid assistive limb (HAL) exoskeleton (84). Monitoring through the study and at 1-year follow-up, found no recurrence of pain and nor the need for the pain relief medications since the initiation of exoskeleton training. Although this study utilized the exoskeleton with BWSTT, compared with the two large-scale studies, with 24 (17) and 26 (11) training sessions, may suggest that the benefits of exoskeleton training on the reduction of neuropathic pain to require longer training durations. Future studies should focus on minimizing selection bias in the terms of psychological states and individual pain thresholds.

## Spasticity

A common method adopted for the evaluation of spasticity was the Modified Ashworth Scale (MAS) (6, 11, 17, 38, 85). A study by Baunsgaard et al. (17) measured the MAS for antagonistic muscle groups at the hips, knees, and ankles. Their results showed a significant decrease in MAS score in all muscle groups after 12 sessions, and after the completion of a total of 24 sessions of training, with the Ekso GT exoskeleton, compared with baseline. This decrease of spasticity, however, was absent at 4 weeks of follow-up after the end of the training program. Juszczak et al. (11) measured MAS scores as the same muscle groups as Baunsgaard et al., pre- and post-trial. They reported that, although 12 out of 45 patients were found to have a decrease in spasticity, 5 were found to have increased spasticity. However, the self-reported data from the same study participants showed a significant decrease in spasticity. Despite its wide adoption, limitations, such as subjective evaluation and simple scoring, should be recognized in the use of MAS (85).

Additionally, in a study with only one training session with the Ekso exoskeleton (6), a significant decrease in spasticity was found among its 21 SCI participants across multiple metrics for spasticity, such as subjective measurements with the numerical rating scale (NRS), the Penn Spasm Frequency Scale (PSFS), and semi-quantitative documentations with MAS. The study by Khan et al. (37) used the Spinal Cord Assessment Tool for Spasticity (SCATS) for the assessment of spasticity in 12 SCI individuals. Their data showed two uniquely different pattern of changes in subjects with low- and high-spasticity scores initially, despite the smallest meaningful change remaining unknown. We suggest that future studies should standardize the timing of assessment with reference to the training period. Data extraction of 11 articles found on this topic is listed in Table 6.

**Table 6:** Spasticity.

**References**	**Intervention (Exoskeleton)**	**No. of training sessions**	**Duration of each session (mins)**	**ASIA Grade (No. of participants)**	**Point when measurements were taken**	**Metrics used**	**Presence of any controls**
Bach Baunsgaard et al. (26)	Ekso GT	24	31.5	36A+B/3C/16D (52)	First and last session, midway through program and follow up	MAS applied to hip flexors/ extensors, knee flexors and extensors, ankle dorsi-flexors/ plantar flexors	No
Ekelem and Goldfarb (85)	Indego with functional electrical stimulation (FES)	^*^	^*^	2B (2)	Before and after each session	MAS applied bilaterally in the hip flexors, hip extensors, knee flexor, knee extensor, hip adductors, hip abductors, ankle dorsiflexor, and ankle plantar flexor muscles.	No
Esquenazi et al. (68)	ReWalk	24	75–90	Chronic thoracic motor-complete SCI (12)^*^	Before and after each session	Patient self-reporting	No
Juszczak et al. (11)	Indego	26	N/A	30A/5B/10C (45)	Before and after each session	MAS applied to hip, knee and ankle flexors	No
						Patient self-reporting	
Khan et al. (37)	ReWalk	Around 52		6A/2B/3C/1D		Spinal cord Assessment Tool for Spasticity (SCATS)	No
Kim et al. (38)	Hyundai Medical Exoskeleton [H-MEX]	30	60	7A/1B/2C (10)	Before and after training program	MAS applied bilaterally in the knee flexor, knee extensor, ankle dorsiflexor, and ankle plantar flexor muscles.	No
Kozlowski et al. (40)	Ekso	20	120	3A/1B/3C (7)	^*^	Patient self-reporting	No
Kressler et al. (42)	Ekso	18	60	3A	First and last session, midway through training	Spinal Cord Assessment Tool for Spastic Reflexes	No
						Spinal reflex excitability was assessed by normalising the maximal soleus H-reflex/M-wave	
Platz et al. (69)	ReWalk	28–35	60	6A/1C (7)	^*^	Resistance to Passive Movement Scale (REPAS)	
Stampacchia et al. (6)	Ekso	1	40	12A/2B/7D	Before and after the session	MAS applied to hip, knee and ankle flexors	No
						Spasms quantified using Penn Spasm Frequency Scale (PSFS)	
						NRS	
Zeilig et al. (51)	ReWalk	13.7	50	A/B (6)	After training program	Satisfaction questionnaire	No

* Details not provided; VAS, visual analogue scale; MAS, Modified Ashworth Scale; NRS, numerical rating scale (0–10); ^*^, Details not provided; ASIA LEMS, American Spinal Cord Injury Assessment lower extremity motor score; ISNCSCI LEMS, International Standards for Neurological Classification of Spinal Cord Injury lower extremity motor score.

## Quality of Life

Studies evaluated the changes in QOL of SCI individuals using different surveys. In the feasibility study by Benson et al. (27), an Assistive Technology Device Predisposition Assessment (ATD-PA) questionnaire was used to evaluate the changes in the QOL of 10 SCI individuals with ReWalk exoskeleton. A small improvement was seen in the QOL subsection of the questionnaire. The rest of the results showed that devices did not meet patients' expectations by study endpoint, with moderate scores for the hypothetical use of exoskeletons in the community.

The study by Baunsgaard et al. (17), using the International SCI Basic Data to assess QOL, found a significant increase in satisfaction with life only in the chronically injured group, but not in the recently injured group, from baseline to the end of the study, as well as to follow-up. On the other hand, the study by Juszczak et al. (11) using the Satisfaction with Life Scale (SWLS) found no significant change in the QOL after training with exoskeletons. Data extraction of the 11 articles found on this topic is listed in Table 7.

**Table 7:** Quality of life.

**References**	**Intervention (Exoskeleton)**	**No. of training sessions**	**Duration of each session (mins)**	**ASIA Grade (No. of participants)**	**Point when measurements were taken**	**Metrics used**	**Presence of any controls**
Bach Baunsgaard et al. (26)	Ekso GT	24	31.5	36A+B/3C/16D (52)	First, last session and follow up	International SCI BASIC Data Sets	No
Benson et al. (27)	ReWalk	10	60	7A/3C (10)	Before and after training program	ATD-PA questionnaire	No
Cahill et al. (86)	Ekso	*	*	* (4)	N/A	Interviews	No
Hartigan et al. (35)	Rewalk	48	60	* (11)	N/A	Interviews	No
Juszczak et al. (11)	Indego	26	N/A	30A/5B/10C	Before each session	SWLS	
Kim et al. (38)	Hyundai Medical Exoskeleton [H-MEX]	30	60	7A/1B/2C (10)	Before and after training program	SF-36v2	No
Kinnett-Hopkins et al. (87)	Mainly Ekso, Indego, ReWalk	N/A	N/A	Majority incomplete (28)	N/A	Focus groups	No
Platz et al. (69)	ReWalk	28–35	60	6A/1C (7)	First, last session and follow up	SF-12v2	No
Raab et al. (71)	Rewalk	120	60	1C (1)	Before and after training program	SF-36	No
Thomassen et al. (88)	Ekso	N/A	N/A	A/C (3)	N/A	Interviews	No
Wolff et al. (89)	N/A	N/A	N/A	N/A	N/A	Online survey	N/A

*SF-12v2, Short Form survey version 2 (12 questions); SF-36v2, Short Form survey version 2 (36 questions); SWLS, Satisfactions with Life Scale; ATD-PA, Assistive Technology Device Predisposition Assessment*.

## Improving Independence of Individuals With SCI

### Independence

One method used to evaluate the independence of individuals with SCI in the community and home setting, is the SCIM. The SCIM evaluates the self-care, respiration, and sphincter management, and mobility (90). Among studies that used the SCIM in its various iterations (17, 69, 91), only a study by Baunsgaard et al. (17) showed a significant improvement in the independence measure, for both recently and chronically injured SCI individuals, after 24 sessions. These results were not replicated in the study by Platz et al. (69), where no systemic change was noted after 28–35 training sessions. Data extraction of 6 articles on this topic is listed in Table 8.

**Table 8:** Independence and community use of exoskeletons.

**References**	**Intervention (Exoskeleton)**	**No. of training sessions**	**Duration of each session (mins)**	**ASIA Grade (No. of participants)**	**Point when measurements were taken**	**Metrics used**	**Presence of any controls**
Bach Baunsgaard et al. (26)	Ekso, Ekso GT	16	*	36A/B/16C (52)	Before and after training program and at 4 weeks of follow-up	WISCI II	No
Baunsgaard et al. (17)	Ekso GT	24	31.5	36A+B/3C/16D (52)	First, last session and follow up	SCIM III	No
Platz et al. (69)	ReWalk	28–35	60	6A/1C (7)	*	SCIM	No
Stampacchia et al. (6)	Ekso	1	40	12A/2B/7D	During the session	SCIM II	No
Tsai et al. (92)	Ekso GT with physical and occupational therapy	4.2	49.7	1A/1B/5C/3D	Before and after training program	FIM	2A/2B/8C/8D (20) with physical therapy and occupational therapy (retrospective)
van Dijsseldonk et al. (93)	ReWalk Personal 6.0	8weeks	NA	13A/1B	N/A	Exoskeleton use from software and logbook	No
						Quebec User Evaluation of satisfaction with assistive Technology (D-QUEST)	
						System Usability Scale (SUS)	
Xiang et al. (91)	AIDER	20	30	22A/6B	First and last session, midway through training program	Hoffer walking ability grade	No
						SCIM III	
						WISCI II	

*SCIM, Spinal Cord Independence Measure; FIM, Functional Independence Measure; WISCI II, Walking Index for SCI II*.

### Applicability in Practical Environments

One important aspect of exoskeleton application in daily life is the safe ambulatory speed of its users. It has been shown that an ambulatory speed of 0.49 m/s is necessary for general ambulation across a road junction with traffic lights (94). In a systematic review by Louie et al. (95), it was found that the mean exoskeleton walking speed of persons with complete SCI was 0.26 m/s. Four variables were found in the majority of studies which might influence gait speed within an exoskeleton, such as age, injury duration, injury level, and the number of training sessions. Another aspect of daily use of exoskeletons relates to the ease of donning and doffing the device, Bryce et al. (96) have suggested that individuals should be able to don and doff an exoskeleton independently in 5 min or less for it to be considered viable for community use. Although this requirement was not achieved by all participants in the study conducted by Tefertiller et al. (97) 84% of the study participants could don and doff the exoskeleton by themselves, and the time required decrease from midway assessment to final evaluation, with average don on time being 9:01 min, and doff time being 2:44 min.

In a recent observational study, the study participants, who were deemed to have predefined proficiency with the ReWalk Personal 6.0 exoskeleton, were allowed to use the exoskeleton in their home and community for 8 weeks (93). The study found that most users adopted the device for individual exercise (78%), and the outdoors area was the most common location of use (48%). The overall response by the participants were positive; the main criticism claimed that the necessity for constant supervision by a buddy was a hindrance.

## Discussion

The purpose of this review was to summarize the current available literature on health-related benefits of exoskeleton walking for SCI individuals, and to identify gaps in the current knowledge base. Although much research has been devoted for developing and refining the mechanics and kinetics of exoskeletons, this review has highlighted focus areas in the currently available literature that need to be addressed before a strong conviction can be had on the health benefits of exoskeleton use.

### Knowledge Gaps Identified by This Scoping Review

This review has revealed that SCI research is still limited in the area of CVS benefits of longer-term exoskeleton use. Although we found 31 studies on this topic, the implementation of a wide range of metrics in measuring CVS health has resulted in vastly heterogeneous studies that are difficult to compare. This heterogeneity is prevalent in the variety of study methods utilized within existing studies, where the amount of training sessions can range from 1 (6) to 60 (58). Variations in the duration of training per session, and selected time points for data collection have also been noted (Tables 1–8). As illustrated by the case of neuropathic pain, the longer periods of training with the exoskeletons may be required to elicit its associated health benefits, and the floor effect has to be considered when choosing suitable subjects for study evaluation (84). Other variables affecting the observed results include rest frequency and duration.

Investigations on the effects of exoskeleton use on BMD and body composition have not received sufficient emphasis. We identified only three studies with a small number of patients which have attempted to elucidate the longer-term benefits with regard to the preservation of BMD after training (38, 63, 68). In addition, we found only three studies investigating changes in body mass composition (63, 74, 75). Given the promising results suggested by the existing data, it may be worthwhile to continue exploring on the benefits of exoskeletons in this area in the future.

The applicability of exoskeleton toward independence and functional gain is another area which we have identified as having significant knowledge gaps. Several studies have attempted to utilize independence measures, and walking indices as proxies for community acceptance of this type of technology. Within the subtopic of the viability of exoskeleton use in the community setting, many studies have utilized methods relying on theoretical situations, *via* questionnaires, to tackle this dilemma. Additionally, it is intuitive that on a practical level, combining home modifications and exoskeleton use can maximize functional gain and reduce risks. Therefore, it may be more useful for upcoming studies to directly evaluate the use of these devices at home, as was done by van Dijsseldonk et al. (93), for a more accurate appreciation of exoskeleton use beyond the controlled clinical environment.

### Recommendations

In the terms of methodology, future studies may benefit from restricting their study populations into cohorts with a narrower range of injury severities and the neurological levels of SCI. Although this may result in a decrease in the size of the study cohort, this can strengthen any findings identified for secondary complications, such as neuropathic pain, bowel, and motor function, which vary greatly depending on the level and severity of the SCI. Factors beyond the type of SCI should also be controlled, as factors such as prior athleticism or hand-eye coordination, can also have an impact on the results demonstrated from exoskeleton training. Increased training time with the exoskeletons may help uncover the benefits in health parameters, such as BMD and body composition, which may require much longer periods of exoskeleton use for the physiological changes to become measurable. One review has provided a recommended duration of 2 h per session, with at least 20 sessions (19), due to the observation that increases in gait speed are correlated with training time (95). This observation is likely to expand beyond gait speed to other secondary complications. Using objective outcome measures for urination, such as urodynamic studies can improve the consistency of study results.

When choosing outcome metrics, we recommend that parameters selected not only need to be able to consistently reflect changes on the development of secondary complications but should also be widely available. For example, in the case of CVS health, adopting PCI may be more advisable than performing gas analysis due to varying access and constraints to resources (32, 62). Patient surveys and reporting, which were utilized by many individual studies, are useful in identifying the impact of the exoskeleton use on factors, such as neuropathic pain and bowel dysfunction, and also allow researchers to evaluate the effects of the use of exoskeleton from the end user perspective. However, it may be more accurate to opt for more measurable metrics, such as the amount of pain killer medication consumed, defecation time, which can aid in making comparison across studies, since their methodologies can be verified and replicated more reliably, and placebo effects can be accounted for. Additionally, data obtained from bread and butter physical tests, such as the 6-min walking test (6MWT), can be further enhanced with the use of accessory devices, to provide greater insight into the functional status of the patient (98).

In this review, we have noticed significant heterogeneity in the timepoint(s) of data collection, irrespective of the parameter, which has made cross-trial comparisons difficult. Some studies obtained their measurements only when the exoskeleton users could ambulate with their exoskeletons continuously (25), while others did not measure any results in the follow-up after the endpoint of the study. Additionally, it will be useful for the studies to measure the same parameters at baseline and study endpoint, for a comparison to be made within the individual subjects. Longer follow-up periods are needed to examine the lasting effects or rebound of symptoms after the cessation of exoskeleton training.

Finally, an important question to answer is the superiority of the exoskeleton over other forms of ambulation for SCI individuals, as done by another trial that has investigated the effect of exoskeletons on patients with stroke (99). It is advisable that future case-control studies compare exoskeleton use directly with other modes of ambulation, such as gait orthoses or BWSTT, as done by Huang et al. (66). Doing so may also allow a comparison of cost-effectiveness of the different devices, beyond that of secondary health benefits. One example would be a case-control study evaluating the effect of exoskeleton training, vs. activity-based training (ABT) (31). Although this study found meaningful clinical change in CVS parameters as early as 6 weeks, it was also discovered that the stimulation of standing in an ABT was also able to achieve a comparable CVS response to exoskeleton training. This may suggest a more cost-effective method of achieving the same CVS benefits.

## Limitations

The scope of this review is only limited to overground, lower limb, and powered robotic exoskeletons. Therefore, the criticisms on study methods do not apply to studies conducted on the other forms of exoskeletons, though we believe that the recommendations made can be universally applied.

## Conclusion

In summary, overground exoskeletons have much potential in maintaining good health and improving the QOL in individuals with SCI. Existing data suggest noticeable benefits across different types of secondary health complications due to prolonged immobilization, as well as a positive response by individual with SCI for the potential chance to regain their previous roles in the community. This scoping review has identified several focus areas in the need of further investigation, such as CVS health, BMD, body composition changes, and independence and functional applications for exoskeletons. We have identified pitfalls, such as heterogeneous methodologies, disparate study populations, and dissimilar training programs, which need to be overcome in future studies. By improving the body of evidence supporting the positive health benefits of exoskeleton use, health policymakers and healthcare professions may better facilitate its mainstream application for the health maintenance of individuals with SCI (29, 46).

## Data Availability Statement

The original contributions presented in the study are included in the article/supplementary material, further inquiries can be directed to the corresponding author.

## Author Contributions

The manuscript was prepared by CY and PK. KC, YW, and C-YL participated in the editing and revisions of the manuscript. All authors contributed to the article and approved the submitted version.

## Conflict of Interest

The authors declare that the research was conducted in the absence of any commercial or financial relationships that could be construed as a potential conflict of interest.

## Publisher's Note

All claims expressed in this article are solely those of the authors and do not necessarily represent those of their affiliated organizations, or those of the publisher, the editors and the reviewers. Any product that may be evaluated in this article, or claim that may be made by its manufacturer, is not guaranteed or endorsed by the publisher.

## References

[1] Jensen MP Truitt AR Schomer KG Yorkston KM Baylor C Molton IR. Frequency and age effects of secondary health conditions in individuals with spinal cord injury: a scoping review. Spinal Cord. (2013) 51:882–92. 10.1038/sc.2013.112 24126851

[2] Sezer N Akkuş S Ugurlu FG. Chronic complications of spinal cord injury. World J Orthop. (2015) 6:24–33. 10.5312/wjo.v6.i1.24 25621208 PMC4303787

[3] Negrini S Imperio G Villafañe JH Negrini F Zaina F. Systematic reviews of physical and rehabilitation medicine cochrane contents. Part 1. disabilities due to spinal disorders and pain syndromes in adults. Eur J Phys Rehabil Med. (2013) 49:597–609. 24084418

[4] Karimi MT. Evidence-Based evaluation of physiological effects of standing and walking in individuals with spinal cord injury. Iran J Med Sci. (2011) 36:242–53. 23115408 PMC3470285

[5] Kohrt WM Barry DW Schwartz RS. Muscle forces or gravity: what predominates mechanical loading on bone? Med Sci Sports Exerc. (2009) 41:2050–5. 10.1249/MSS.0b013e3181a8c717 19812511 PMC3037021

[6] Stampacchia G Rustici A Bigazzi S Gerini A Tombini T Mazzoleni S. Walking with a powered robotic exoskeleton: subjective experience, spasticity and pain in spinal cord injured persons. NeuroRehabilitation. (2016) 39:277–83. 10.3233/NRE-161358 27372363

[7] Craven BC Giangregorio LM Alavinia SM Blencowe LA Desai N Hitzig SL . Evaluating the efficacy of functional electrical stimulation therapy assisted walking after chronic motor incomplete spinal cord injury: effects on bone biomarkers and bone strength. J Spinal Cord Med. (2017) 40:748–58. 10.1080/10790268.2017.1368961 28929919 PMC5778938

[8] Lajeunesse V Vincent C Routhier F Careau E Michaud F. Exoskeletons' design and usefulness evidence according to a systematic review of lower limb exoskeletons used for functional mobility by people with spinal cord injury. Disabil Rehabil Assist Technol. (2016) 11:535–47. 10.3109/17483107.2015.1080766 26340538

[9] Lünenburger L Colombo G Riener R Dietz V. Biofeedback in gait training with the robotic orthosis lokomat. Conf Proc IEEE Eng Med Biol Soc. (2004) 2004:4888–91. 10.1109/IEMBS.2004.1404352 17271408

[10] Harvey LA Lin CW Glinsky JV De Wolf A. The effectiveness of physical interventions for people with spinal cord injuries: a systematic review. Spinal Cord. (2009) 47:184–95. 10.1038/sc.2008.100 18725889

[11] Juszczak M Gallo E Bushnik T. Examining the effects of a powered exoskeleton on quality of life and secondary impairments in people living with spinal cord injury. Top Spinal Cord Injury Rehabil. (2018) 24:336–42. 10.1310/sci17-00055 30459496 PMC6241230

[12] Ditunno PL Patrick M Stineman M Ditunno JF. Who wants to walk? Preferences for recovery after sci: a longitudinal and cross-sectional study. Spinal Cord. (2008) 46:500–6. 10.1038/sj.sc.3102172 18209742

[13] Anderson KD. Targeting recovery: priorities of the spinal cord-injured population. J Neurotrauma. (2004) 21:1371–83. 10.1089/neu.2004.21.1371 15672628

[14] Miller LE Zimmermann AK Herbert WG. Clinical effectiveness and safety of powered exoskeleton-assisted walking in patients with spinal cord injury: systematic review with meta-analysis. Med Devices (Auckl). (2016) 9:455–66. 10.2147/MDER.S103102 27042146 PMC4809334

[15] Fisahn C Aach M Jansen O Moisi M Mayadev A Pagarigan KT . The effectiveness and safety of exoskeletons as assistive and rehabilitation devices in the treatment of neurologic gait disorders in patients with spinal cord injury: a systematic review. Global Spine J. (2016) 6:822–41. 10.1055/s-0036-1593805 27853668 PMC5110426

[16] Mekki M Delgado AD Fry A Putrino D Huang V. Robotic rehabilitation and spinal cord injury: a narrative review. Neurotherapeutics. (2018) 15:604–17. 10.1007/s13311-018-0642-3 29987763 PMC6095795

[17] Baunsgaard CB Nissen UV Brust AK Frotzler A Ribeill C Kalke YB . Exoskeleton gait training after spinal cord injury: an exploratory study on secondary health conditions. J Rehabil Med. (2018) 50:806–13. 10.2340/16501977-2372 30183055

[18] Duddy D Doherty R Connolly J McNally S Loughrey J Faulkner M. The effects of powered exoskeleton gait training on cardiovascular function and gait performance: a systematic review. Sensors (Basel). (2021) 21:3207. 10.3390/s21093207 34063123 PMC8124924

[19] Contreras-Vidal JL N AB Brantley J Cruz-Garza JG He Y Manley Q . Powered exoskeletons for bipedal locomotion after spinal cord injury. J Neural Eng. (2016) 13:031001. 10.1088/1741-2560/13/3/031001 27064508

[20] Arksey H O'Malley L. Scoping studies: towards a methodological framework, Int. J. Soc. Res. Methodol. (2005) 8:19–32. 10.1080/1364557032000119616

[21] Garshick E Kelley A Cohen SA Garrison A Tun CG Gagnon D . A prospective assessment of mortality in chronic spinal cord injury. Spinal Cord. (2005) 43:408–16. 10.1038/sj.sc.3101729 15711609 PMC1298182

[22] Nash MS. Exercise as a health-promoting activity following spinal cord injury. J Neurol Phys Ther. (2005) 29:87–103:6. 10.1097/01.NPT.0000282514.94093.c6 16386165

[23] Sisto SA Evans N. Activity and fitness in spinal cord injury: review and update. Curr Phys Med Rehabil Rep. (2014) 2:147–57. 10.1007/s40141-014-0057-y

[24] Arazpour M Bani MA Hutchins SW Jones RK. The physiological cost index of walking with mechanical and powered gait orthosis in patients with spinal cord injury. Spinal Cord. (2013) 51:356–9. 10.1038/sc.2012.162 23247013

[25] Asselin P Knezevic S Kornfeld S Cirnigliaro C Agranova-Breyter I Bauman WA . Heart rate and oxygen demand of powered exoskeleton-assisted walking in persons with paraplegia. J Rehabil Res Dev. (2015) 52:147–58. 10.1682/JRRD.2014.02.0060 26230182

[26] Bach Baunsgaard C Vig Nissen U Katrin Brust A Frotzler A Ribeill C Kalke YB . Gait training after spinal cord injury: safety, feasibility and gait function following 8 weeks of training with the exoskeletons from ekso bionics. Spinal Cord. (2018) 56:106–16. 10.1038/s41393-017-0013-7 29105657

[27] Benson I Hart K Tussler D van Middendorp JJ. Lower-Limb exoskeletons for individuals with chronic spinal cord injury: findings from a feasibility study. Clin Rehabil. (2016) 30:73–84. 10.1177/0269215515575166 25761635

[28] Escalona MJ Brosseau R Vermette M Comtois AS Duclos C Aubertin-Leheudre M . Cardiorespiratory demand and rate of perceived exertion during overground walking with a robotic exoskeleton in long-term manual wheelchair users with chronic spinal cord injury: a cross-sectional study. Ann Phys Rehabil Med. (2018) 61:215–23. 10.1016/j.rehab.2017.12.008 29371106

[29] Evans N Hartigan C Kandilakis C Pharo E Clesson I. Acute cardiorespiratory and metabolic responses during exoskeleton-assisted walking overground among persons with chronic spinal cord injury. Top Spinal Cord Inj Rehabil. (2015) 21:122–32. 10.1310/sci2102-122 26364281 PMC4568093

[30] Evans R Shackleton C West S Rauch L Derman W Albertus Y. Improvements in cardiovascular efficiency over 24-weeks of robotic locomotor training in persons with sci. Arch Phys Med Rehabil. (2019) 100:e184. 10.1016/j.apmr.2019.10.067

[31] Evans RW Shackleton C West S Derman W Rauch HL Baalbergen E . Robotic locomotor training leads to cardiovascular changes in individuals with incomplete spinal cord injury over a 24-week rehabilitation period: a randomized controlled pilot study. Arch Phys Med Rehabil. (2021) 102:1447–56. 10.1016/j.apmr.2021.03.018 33839105

[32] Farris RJ Quintero HA Murray SA Ha KH Hartigan C Goldfarb M. A preliminary assessment of legged mobility provided by a lower limb exoskeleton for persons with paraplegia. IEEE Trans Neural Syst Rehabil Eng. (2014) 22:482–90. 10.1109/TNSRE.2013.2268320 23797285 PMC4476394

[33] Faulkner J Martinelli L Cook K Stoner L Ryan-Stewart H Paine E . Effects of robotic-assisted gait training on the central vascular health of individuals with spinal cord injury: a pilot study. J Spinal Cord Med. (2019) 44:299–305. 10.1080/10790268.2019.1656849 31525137 PMC7952073

[34] Gorgey A Wade R Villadelgado L Lavis REKT. Exoskeleton training improves parameters of physical activity in a person with tetraplegia. Arch Phys Med Rehabil. (2016) 97:e132. 10.1016/j.apmr.2016.08.411

[35] Hartigan C Goldfarb M Farris R Ha K Murray S. Newly developed robotic exoskeleton that provides mobility and option for functional electrical stimulation. Arch Phys Med Rehabil. (2013) 94:e18–e9. 10.1016/j.apmr.2013.08.065

[36] Jang YC Park HK Han JY Choi IS Song MK. Cardiopulmonary function after robotic exoskeleton-assisted over-ground walking training of a patient with an incomplete spinal cord injury: case report. Medicine (Baltimore). (2019) 98:e18286. 10.1097/MD.0000000000018286 31852105 PMC6922438

[37] Khan AS Livingstone DC Hurd CL Duchcherer J Misiaszek JE Gorassini MA . Retraining walking over ground in a powered exoskeleton after spinal cord injury: a prospective cohort study to examine functional gains and neuroplasticity. J Neuroeng Rehabil. (2019) 16:145. 10.1186/s12984-019-0585-x 31752911 PMC6868817

[38] Kim HS Park JH Lee HS Lee JY Jung JW Park SB . Effects of wearable powered exoskeletal training on functional mobility, physiological health and quality of life in non-ambulatory spinal cord injury patients. J Korean Med Sci. (2021) 36:e80. 10.3346/jkms.2021.36.e80 33783145 PMC8007419

[39] McIntosh K Charbonneau R Bensaada Y Bhatiya U Ho C. The safety and feasibility of exoskeletal-assisted walking in acute rehabilitation after spinal cord injury. Arch Phys Med Rehabil. (2020) 101:113–20. 10.1016/j.apmr.2019.09.005 31568761

[40] Kozlowski AJ Bryce TN Dijkers MP. Time and effort required by persons with spinal cord injury to learn to use a powered exoskeleton for assisted walking. Top Spinal Cord Inj Rehabil. (2015) 21:110–21. 10.1310/sci2102-110 26364280 PMC4568092

[41] Kressler J Domingo A. Cardiometabolic challenges provided by variable assisted exoskeletal versus overground walking in chronic motor-incomplete paraplegia: a case series. J Neurol Phys Ther. (2019) 43:128–35. 10.1097/NPT.0000000000000262 30883500

[42] Kressler J Thomas CK Field-Fote EC Sanchez J Widerström-Noga E Cilien DC . Understanding therapeutic benefits of overground bionic ambulation: exploratory case series in persons with chronic, complete spinal cord injury. Arch Phys Med Rehabil. (2014) 95:1878–87.e4. 10.1016/j.apmr.2014.04.026 24845221

[43] Kressler J Wymer T Domingo A. Respiratory, cardiovascular and metabolic responses during different modes of overground bionic ambulation in persons with motor-incomplete spinal cord injury: a case series. J Rehabil Med. (2018) 50:173–80. 10.2340/16501977-2281 29068039

[44] Kwon SH Lee BS Lee HJ Kim EJ Lee JA Yang SP . Energy efficiency and patient satisfaction of gait with knee-ankle-foot orthosis and robot (rewalk)-assisted gait in patients with spinal cord injury. Ann Rehabil Med. (2020) 44:131–41. 10.5535/arm.2020.44.2.131 32392652 PMC7214138

[45] Maher JL Baunsgaard CB van Gerven J Palermo AE Biering-Sorensen F Mendez A . Differences in acute metabolic responses to bionic and nonbionic ambulation in spinal cord injured humans and controls. Arch Phys Med Rehabil. (2020) 101:121–9. 10.1016/j.apmr.2019.07.014 31465760

[46] Pérez-Nombela S del-Ama AJ Asín-Prieto G Piñuela-Martín E Lozano-Berrio V Serrano-Muñoz D . editors. Physiological Evaluation of Different Control Modes of Lower Limb Robotic Exoskeleton H2 in Patients With Incomplete Spinal Cord Injury. Cham: Springer International Publishing (2017).

[47] Sale P Russo EF Russo M Masiero S Piccione F Calabrò RS . Effects on mobility training and de-adaptations in subjects with spinal cord injury due to a wearable robot: a preliminary report. BMC Neurol. (2016) 16:12. 10.1186/s12883-016-0536-0 26818847 PMC4730780

[48] Spungen AM Asselin PK Fineberg DB Kornfeld SD Harel NY. Exoskeletal-assisted walking for persons with motor-complete paraplegia. NATO Science and Technology Organization. (2013) 15–7.

[49] Wu CH Mao HF Hu JS Wang TY Tsai YJ Hsu WL. The effects of gait training using powered lower limb exoskeleton robot on individuals with complete spinal cord injury. J Neuroeng Rehabil. (2018) 15:14. 10.1186/s12984-018-0355-1 29506530 PMC5838988

[50] Xiang XN Zong HY Ou Y Yu X Cheng H Du CP . Exoskeleton-Assisted walking improves pulmonary function and walking parameters among individuals with spinal cord injury: a randomized controlled pilot study. J Neuroeng Rehabil. (2021) 18:86. 10.1186/s12984-021-00880-w 34030720 PMC8146689

[51] Zeilig G Weingarden H Zwecker M Dudkiewicz I Bloch A Esquenazi A. Safety and tolerance of the rewalk™ exoskeleton suit for ambulation by people with complete spinal cord injury: a pilot study. J Spinal Cord Med. (2012) 35:96–101. 10.1179/2045772312Y.0000000003 22333043 PMC3304563

[52] Ainsworth BE Haskell WL Leon AS Jacobs DR Jr Montoye HJ . Compendium of physical activities: classification of energy costs of human physical activities. Med Sci Sports Exerc. (1993) 25:71–80. 10.1249/00005768-199301000-00011 8292105

[53] Collins EG Gater D Kiratli J Butler J Hanson K Langbein WE. Energy cost of physical activities in persons with spinal cord injury. Med Sci Sports Exerc. (2010) 42:691–700. 10.1249/MSS.0b013e3181bb902f 19952846

[54] Garber CE Blissmer B Deschenes MR Franklin BA Lamonte MJ Lee IM . American college of sports medicine position stand. Quantity and quality of exercise for developing and maintaining cardiorespiratory, musculoskeletal, and neuromotor fitness in apparently healthy adults: guidance for prescribing exercise. Med Sci Sports Exerc. (2011) 43:1334–59. 10.1249/MSS.0b013e318213fefb 21694556

[55] Goosey-Tolfrey VL Paulson TA Tolfrey K Eston RG. Prediction of peak oxygen uptake from differentiated ratings of perceived exertion during wheelchair propulsion in trained wheelchair sportspersons. Eur J Appl Physiol. (2014) 114:1251–8. 10.1007/s00421-014-2850-9 24610244

[56] van der Scheer JW Hutchinson MJ Paulson T Martin Ginis KA Goosey-Tolfrey VL. Reliability and validity of subjective measures of aerobic intensity in adults with spinal cord injury: a systematic review. Pm r. (2018) 10:194–207. 10.1016/j.pmrj.2017.08.440 28867664

[57] Sale P Russo EF Scarton A Calabrò RS Masiero S Filoni S. Training for mobility with exoskeleton robot in spinal cord injury patients: a pilot study. Eur J Phys Rehabil Med. (2018) 54:745–51. 10.23736/S1973-9087.18.04819-0 29517187

[58] Knezevic S Asselin PK Cirnigliaro CM Kornfeld S Emmons RR Spungen AM. Oxygen uptake during exoskeletal-assisted walking in persons with paraplegia. Arch Phys Med Rehabil. (2020) 102:185– 10.1016/j.apmr.2020.08.025 33181116

[59] Hicks AL. Locomotor training in people with spinal cord injury: is this exercise? Spinal Cord. (2021) 59:9–16. 10.1038/s41393-020-0502-y 32581307

[60] Baden D Warwick-Evans L Lakomy J. Am I Nearly There? The effect of anticipated running distance on perceived exertion and attentional focus. J Sport Exerc Psychol. (2004) 27:215–31. 10.1123/jsep.26.2.215

[61] Fineberg DB Asselin P Harel NY Agranova-Breyter I Kornfeld SD Bauman WA . Vertical ground reaction force-based analysis of powered exoskeleton-assisted walking in persons with motor-complete paraplegia. J Spinal Cord Med. (2013) 36:313–21. 10.1179/2045772313Y.0000000126 23820147 PMC3758528

[62] Rukina NN Kuznetsov AN Borzikov VV Komkova OV Belova AN. Principles of efficiency and safety assessment in using exoskeletons for patients with lower limb paralyses. Sovremennye Tehnol Med. (2016) 8:231–40. 10.17691/stm2016.8.4.28

[63] Karelis AD Carvalho LP Castillo MJ Gagnon DH Aubertin-Leheudre M. Effect on body composition and bone mineral density of walking with a robotic exoskeleton in adults with chronic spinal cord injury. J Rehabil Med. (2017) 49:84–7. 10.2340/16501977-2173 27973679

[64] Vlachopoulos C Aznaouridis K Stefanadis C. Prediction of cardiovascular events and all-cause mortality with arterial stiffness: a systematic review and meta-analysis. J Am Coll Cardiol. (2010) 55:1318–27. 10.1016/j.jacc.2009.10.061 20338492

[65] Astorino TA Bediamol N Cotoia S Ines K Koeu N Menard N . Verification testing to confirm Vo_2_Max attainment in persons with spinal cord injury. J Spinal Cord Med. (2019) 42:494–501. 10.1080/10790268.2017.1422890 29355464 PMC6718936

[66] Huang Q Yu L Gu R Zhou Y Hu C. Effects of robot training on bowel function in patients with spinal cord injury. J Phys Ther Sci. (2015) 27:1377–8. 10.1589/jpts.27.1377 26157223 PMC4483401

[67] Hamid R Averbeck MA Chiang H Garcia A Al Mousa RT Oh SJ . Epidemiology and pathophysiology of neurogenic bladder after spinal cord injury. World J Urol. (2018) 36:1517–27. 10.1007/s00345-018-2301-z 29752515

[68] Esquenazi A Talaty M Packel A Saulino M. The rewalk powered exoskeleton to restore ambulatory function to individuals with thoracic-level motor-complete spinal cord injury. Am J Phys Med Rehabil. (2012) 91:911–21. 10.1097/PHM.0b013e318269d9a3 23085703

[69] Platz T Gillner A Borgwaldt N Kroll S Roschka S. Device-training for individuals with thoracic and lumbar spinal cord injury using a powered exoskeleton for technically assisted mobility: achievements and user satisfaction. Biomed Res Int. (2016) 2016:8459018. 10.1155/2016/8459018 27610382 PMC5005562

[70] Chun A Asselin PK Knezevic S Kornfeld S Bauman WA Korsten MA . Changes in bowel function following exoskeletal-assisted walking in persons with spinal cord injury: an observational pilot study. Spinal Cord. (2020) 58:459–66. 10.1038/s41393-019-0392-z 31822808 PMC7145720

[71] Raab K Krakow K Tripp F Jung M. Effects of training with the rewalk exoskeleton on quality of life in incomplete spinal cord injury: a single case study. Spinal Cord Ser Cases. (2016) 2:15025. 10.1038/scsandc.2015.25 28053728 PMC5125066

[72] Stothers L Amanda C Alamro R Williams A Lam T. Demonstration of levator ani emg activity below the level of injury in complete spinal cord Injury (Sci) using over ground robotic exoskeleton walking. J Urol. (2017) 197(4 Suppl. 1):e1260–e1. 10.1016/j.juro.2017.02.2940

[73] Gorgey AS Dudley GA. Skeletal muscle atrophy and increased intramuscular fat after incomplete spinal cord injury. Spinal Cord. (2007) 45:304–9. 10.1038/sj.sc.3101968 16940987

[74] Asselin P Cirnigliaro CM Kornfeld S Knezevic S Lackow R Elliott M . Effect of exoskeletal-assisted walking on soft tissue body composition in persons with spinal cord injury. Arch Phys Med Rehabil. (2020) 102:196–202. 10.1016/j.apmr.2020.07.018 33171129

[75] Cirnigliaro CM Knezevic S Specht A Lombard AT Spungen AM Bauman WA . Decreased total and central adiposity after 100 exoskeletal-assisted walking sessions in persons with chronic spinal cord injury. J Clin Densitomet. (2018) 21:1. 10.1016/j.jocd.2018.05.013

[76] Giangregorio LM Hicks AL Webber CE Phillips SM Craven BC Bugaresti JM . Body weight supported treadmill training in acute spinal cord injury: impact on muscle and bone. Spinal Cord. (2005) 43:649–57. 10.1038/sj.sc.3101774 15968302

[77] Giangregorio LM Webber CE Phillips SM Hicks AL Craven BC Bugaresti JM . Can body weight supported treadmill training increase bone mass and reverse muscle atrophy in individuals with chronic incomplete spinal cord injury? Appl Physiol Nutr Metab. (2006) 31:283–91. 10.1139/h05-036 16770357

[78] Jayaraman A Shah P Gregory C Bowden M Stevens J Bishop M . Locomotor training and muscle function after incomplete spinal cord injury: case series. J Spinal Cord Med. (2008) 31:185–93. 10.1080/10790268.2008.11760710 18581666 PMC2578797

[79] Stewart BG Tarnopolsky MA Hicks AL McCartney N Mahoney DJ Staron RS . Treadmill training-induced adaptations in muscle phenotype in persons with incomplete spinal cord injury. Muscle Nerve. (2004) 30:61–8. 10.1002/mus.20048 15221880

[80] Smith É Carroll Á. Bone mineral density in adults disabled through acquired neurological conditions: a review. J Clin Densitom. (2011) 14:85–94. 10.1016/j.jocd.2010.12.002 21474350

[81] Ashe MC Craven C Eng JJ Krassioukov A. Prevention and treatment of bone loss after a spinal cord injury: a systematic review. Top Spinal Cord Inj Rehabil. (2007) 13:123–45. 10.1310/sci1301-123 22767990 PMC3389041

[82] Gorgey AS. Robotic exoskeletons: the current pros and cons. World J Orthop. (2018) 9:112–9. 10.5312/wjo.v9.i9.112 30254967 PMC6153133

[83] Dijkers M Bryce T Zanca J. Prevalence of chronic pain after traumatic spinal cord injury: a systematic review. J Rehabil Res Dev. (2009) 46:13–29. 10.1682/JRRD.2008.04.0053 19533517

[84] Cruciger O Schildhauer TA Meindl RC Tegenthoff M Schwenkreis P Citak M . Impact of locomotion training with a neurologic controlled hybrid assistive limb (Hal) exoskeleton on neuropathic pain and health related quality of life (hrqol) in chronic sci: a case study (.). Disabil Rehabil Assist Technol. (2016) 11:529–34. 10.3109/17483107.2014.981875 25382234

[85] Ekelem A Goldfarb M. Supplemental stimulation improves swing phase kinematics during exoskeleton assisted gait of sci subjects with severe muscle spasticity. Front Neurosci. (2018) 12:374. 10.3389/fnins.2018.00374 29910710 PMC5992413

[86] Cahill A Ginley OM Bertrand C Lennon O. Gym-Based exoskeleton walking: a preliminary exploration of non-ambulatory end-user perspectives. Disabil Health J. (2018) 11:478–85. 10.1016/j.dhjo.2018.01.004 29500092

[87] Kinnett-Hopkins D Mummidisetty CK Ehrlich-Jones L Crown D Bond RA Applebaum MH . Users with spinal cord injury experience of robotic locomotor exoskeletons: a qualitative study of the benefits, limitations, and recommendations. J Neuroeng Rehabil. (2020) 17:124. 10.1186/s12984-020-00752-9 32917287 PMC7488437

[88] Thomassen GK Jørgensen V Normann B. “Back at the Same Level as Everyone Else”-User Perspectives on Walking with an Exoskeleton, a Qualitative Study. Spinal Cord Ser Cases. (2019) 5:103. 10.1038/s41394-019-0243-3 31871768 PMC6910916

[89] Wolff J Parker C Borisoff J Mortenson WB Mattie J. A survey of stakeholder perspectives on exoskeleton technology. J Neuroeng Rehabil. (2014) 11:169. 10.1186/1743-0003-11-169 25523497 PMC4320449

[90] Anderson KD Acuff ME Arp BG Backus D Chun S Fisher K . United States (Us) multi-center study to assess the validity and reliability of the spinal cord independence measure (Scim Iii). Spinal Cord. (2011) 49:880–5. 10.1038/sc.2011.20 21445081

[91] Xiang XN Ding MF Zong HY Liu Y Cheng H He CQ . The Safety and feasibility of a new rehabilitation robotic exoskeleton for assisting individuals with lower extremity motor complete lesions following spinal cord injury (sci): an observational study. Spinal Cord. (2020) 58:787–94. 10.1038/s41393-020-0423-9 32034295

[92] Tsai CY Delgado AD Weinrauch WJ Manente N Levy I Escalon MX . Exoskeletal-assisted walking during acute inpatient rehabilitation leads to motor and functional improvement in persons with spinal cord injury: a pilot study. Arch Phys Med Rehabil. (2020) 101:607–12. 10.1016/j.apmr.2019.11.010 31891715

[93] van Dijsseldonk RB van Nes IJW Geurts ACH Keijsers NLW. Exoskeleton home and community use in people with complete spinal cord injury. Sci Rep. (2020) 10:15600. 10.1038/s41598-020-72397-6 32973244 PMC7515902

[94] Andrews AW Chinworth SA Bourassa M Garvin M Benton D Tanner S. Update on distance and velocity requirements for community ambulation. J Geriatr Phys Ther. (2010) 33:128–34. 10.1097/JPT.0b013e3181eda321 21155508

[95] Louie DR Eng JJ Lam T. Gait speed using powered robotic exoskeletons after spinal cord injury: a systematic review and correlational study. J Neuroeng Rehabil. (2015) 12:82. 10.1186/s12984-015-0074-9 26463355 PMC4604762

[96] Bryce TN Dijkers MP Kozlowski AJ. Framework for assessment of the usability of lower-extremity robotic exoskeletal orthoses. Am J Phys Med Rehabil. (2015) 94:1000–14. 10.1097/PHM.0000000000000321 26098923

[97] Tefertiller C Hays K Jones J Jayaraman A Hartigan C Bushnik T . Initial outcomes from a multicenter study utilizing the indego powered exoskeleton in spinal cord injury. Top Spinal Cord Inj Rehabil. (2018) 24:78–85. 10.1310/sci17-00014 29434463 PMC5791927

[98] Pollet J Buraschi R Villafañe JH Piovanelli B Negrini S. Gait parameters assessed with inertial measurement unit during 6-minute walk test in people after stroke. Int J Rehabil Res. (2021) 44:358–63. 10.1097/MRR.0000000000000498 34570043

[99] Taveggia G Borboni A Mulé C Villafañe JH Negrini S. Conflicting results of robot-assisted versus usual gait training during postacute rehabilitation of stroke patients: a randomized clinical trial. Int J Rehabil Res. (2016) 39:29–35. 10.1097/MRR.0000000000000137 26512928 PMC4900426

